# The Role of Cardiac Arrest Sonographic Exam (CASE) in Predicting the Outcome of Cardiopulmonary Resuscitation; a Cross-sectional Study

**DOI:** 10.22037/aaem.v9i1.1272

**Published:** 2021-06-28

**Authors:** Babak Masoumi, Reza Azizkhani, Farhad Heydari, Majid Zamani, Mehdi Nasr Isfahani

**Affiliations:** 1 Department of Emergency Medicine, Faculty of Medicine, Isfahan University of Medical Sciences, Isfahan, Iran.

**Keywords:** Heart arrest, Cardiopulmonary resuscitation, Return of Spontaneous Circulation, Ultrasonography

## Abstract

**Introduction:**

Ultrasonography (US) has been suggested as an integral part of resuscitation to identify potentially reversible causes of cardiac arrest (CA). This study aimed to evaluate the association between cardiac activity on ultrasonography during resuscitation and outcome of patients with non-shockable rhythms.

**Methods:**

We conducted a prospective, observational study on adult patients presenting with CA or experiencing CA in the emergency department (ED), and initial non-shockable rhythm. US examination of the sub-xiphoid region was performed during the 10-second interval of rhythm and pulse check and the association of US findings and patients’ outcomes was evaluated.

**Results:**

151 patients with the mean age of 65.32 ± 11.68 years were evaluated (76.2% male). 43 patients (28.5%) demonstrated cardiac activity on the initial US. The rate of asystole in initial rhythm was 58.9% (n=89). Return of spontaneous circulation (ROSC) was achieved in 36 (23.8%) patients, twenty (13.2%) survived to hospital admission and seven (4.6%) survived to hospital discharge. When the cardiac standstill duration increased to six minutes, no patient survived hospital discharge. Potentially reversible causes were detected in 15 cases (9.9%), and four of them survived to hospital discharge. Cardiac activity on first scan was associated with ROSC (OR: 6.86, 95%CI: 2.92-16.09; p < 0.001), survival to hospital admission (OR: 17.80, 95%CI: 3.95–80.17; p < 0.001), and survival to hospital discharge (OR: 17.35, 95%CI: 2.02–148.92; p = 0.001).

**Conclusion:**

In non-traumatic cardiac arrest patients with non-shockable rhythms, bedside US is of great importance in predicting ROSC. The presence of pulseless electrical activity (PEA) rhythm and cardiac activity on initial US were associated with ROSC, survival to hospital admission, and hospital discharge. When the cardiac standstill duration increased to six minutes, no patient survived hospital discharge.

## 1. Introduction:

Patients with cardiac arrest (CA) should be treated using algorithm-based methods such as Basic Life Support (BLS) and Advanced Life Support (ALS). CA with non-shockable rhythms continues to have poor outcomes (1). Compared to those with a shockable rhythm, patients with pulseless electrical activity (PEA) have poorer prognosis (survival rate: 40% vs 6%, respectively) (2). PEA can be sub-divided into electromechanical dissociation (EMD) (true-PEA) and pseudo-EMD (pseudo-PEA) based on the absence or presence of cardiac activity. The survival rate of pseudo-PEA has been reported to be significantly higher than that of true-PEA, while therapeutic strategies in both cases are similar (3). Rapid identification and correction of potentially reversible causes of cardiac arrest in patients with a non-shockable rhythm is an important step for their management (4).

Ultrasonography (US) has been suggested as an integral part of resuscitation to identify potentially reversible causes of CA, such as pericardial tamponade, pulmonary embolism (PE), tension pneumothorax, and hypovolemia (3-5). Additionally, the US may differentiate between false and true PEA, based on the presence or absence of organized cardiac motion. Detection of cardiac motion on ultrasound may be an early sign of return of spontaneous circulation (ROSC), and is a good predictor of survival (5-7).

Using ultrasonography it is possible to differentiate between true asystole and fine ventricular fibrillation, especially when rhythm monitoring is in doubt (e.g., artifacts), with both prognostic and therapeutic implications (8).

While US has been suggested in CA, there has been no protocol to explore exactly how ultrasound should be integrated with ALS. Furthermore, the main point is that ultrasound does not interfere with the chest compressions. Thus, US is safely integrated into the ALS when it is performed in 10s intervals for rhythm assessment and checking carotid pulse (5-8). However, studies have shown that experienced providers may be able to perform US in less than 10s. A few studies have evaluated the association between US during cardiopulmonary resuscitation (CPR) pauses and interruptions in CPR in the emergency department (ED) (9-11).

A systematic review showed that survival to admission rate in patients without cardiac activity on ultrasound termed cardiac standstill was 2.4% (12). Therefore, the chances of survival associated with cardiac standstill are very low (5).

In this study, we evaluated the association between the cardiac activity on ultrasound during resuscitation and outcome of patients with pulseless electrical activity (PEA) or asystole.

## 2. Methods:

***2.1 Study Design and Setting***

This prospective, observational study was conducted from March 2018 to May 2019 in two urban emergency departments (ED) with an Emergency Medicine residency program (Al-Zahra and Kashani Hospitals, Isfahan, Iran). The study protocol conformed to the principles of the Declaration of Helsinki, and ethics approval was obtained from the ethics committee of Isfahan University of Medical Sciences (IR.MUI.REC.1396.2.070). 

**2.2 Participants**

All non-traumatic patients aged at least 18 years presenting to the ED with cardiac arrest (CA) or experiencing CA in the ED, and initial rhythm of asystole or PEA were eligible. Patients with ROSC before ED arrival, brief resuscitation efforts lasting less than four minutes, or failure to undergo US during resuscitation were excluded.

**2.3 Study protocol**

**Figure 1:**
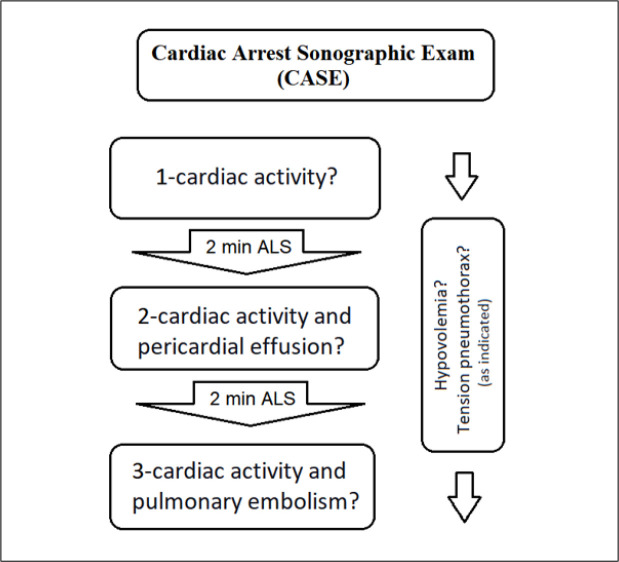
Cardiac Arrest Sonographic Exam (CASE) schematic. ALS: Advanced Life Support

Patients with cardiac arrest were evaluated and managed per ALS guidelines. Our research protocol was a three-step ultrasound (US) protocol that evaluated cardiac activity and reversible causes of CA in non-shockable rhythms. At the beginning of ALS, during the first CPR pauses, the sonographer evaluated and recorded the cardiac activity. During the second pauses, the cardiac activity and pericardial effusion were evaluated and recorded and, if pericardial effusion presented, signs of tamponade such as early diastolic right ventricular collapse were assessed. In the third pauses, cardiac activity and the presence of pulmonary embolism (right ventricular enlargement with left ventricular collapse) were checked and recorded. During CPR, hypovolemia (inferior vena cava [IVC] diameter measurement by US) and tension pneumothorax were evaluated on a case-by-case basis (figure1). Cardiac activity was defined as any visible atrial, valvular, or ventricular movement, excluding movement of blood within the cardiac chambers or isolated valve movement. Treating clinicians were not blinded to the US findings except for the presence or absence of cardiac motion.

All ultrasounds were performed in less than 10 seconds during pauses in resuscitation to determine the cardiac rhythm and pulse checks. To obtain ultrasonic images, CPR did not stop. To avoid any interruption with CPR, the treating emergency physician notified the sonographer 5 seconds before the pulse checks to prepare for a 10-second pause by positioning the ultrasound probe in the sub-xiphoid region to take the four-chamber view of the heart. Ultrasound images were obtained using a sub-xiphoid (subcostal) view (5, 11, 13). Ultrasound was implemented by the five emergency medicine specialists who had more than 6 years’ experience in emergency echocardiography. US service was provided 24 hours a day, 7 days a week. An ultrasonography device (Philips Affiniti 70) with a curved probe (2–6 MHz) was used in this study. 

***2.4 Data gathering***

Standard forms were used to record patients’ age, gender, out-of-hospital or in-hospital arrest, initial rhythms in the ED, ultrasound findings, resuscitation outcome (ROSC, survival to hospital admission, survival to hospital discharge, and death), and the duration of CPR.

**2.5 Statistical analysis**

All analyses were performed using SPSS 22.0 for Windows (SPSS Inc., Chicago, Illinois, USA). Categorical data were reported in frequency and percentages, while continuous data were reported as mean (standard deviation [SD]) or proportion with 95% confidence interval (95% CI). Baseline differences were evaluated via Fisher’s exact and student t-test analyses, and the chi-square test was used to identify differences between groups of nominal variables. 

Binary logistic regression was first performed to obtain odds ratio estimates with 95% confidence intervals (CI) with p values for the three outcomes of 1) ROSC, 2) survival to hospital admission, and 3) survival to hospital discharge. Interaction between independent variables was assessed in a pairwise fashion for all variables. Test characteristics of sensitivity and specificity, positive and negative predictive values, and accuracy of cardiac standstill for the three outcomes were calculated with 95% CI. Statistical significance was set at P <0.05.

## 3. Results:

***3.1 Baseline characteristics of studied cases***

**Figure 2:**
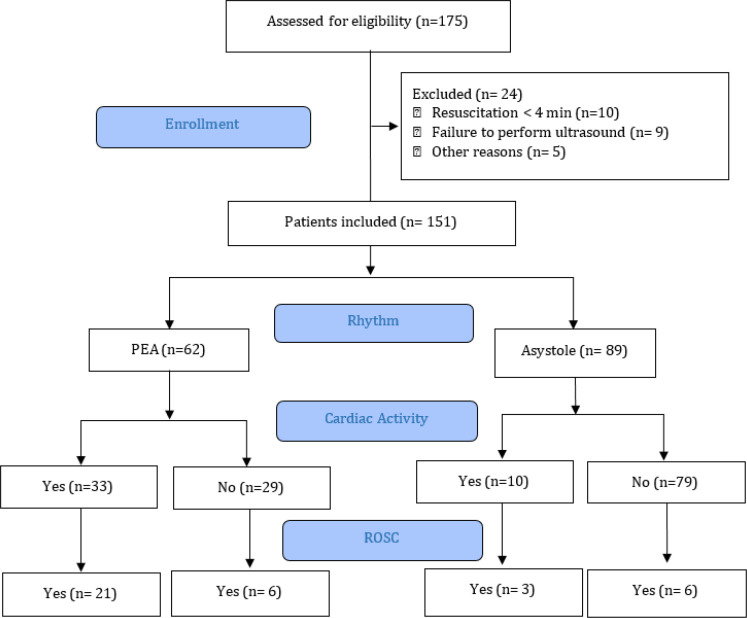
Patients’ flow diagram and outcome

A total of 175 patients were enrolled from March 2018 to May 2019; of which, 151 patients were included in the study and underwent US assessment during cardiac arrest management in the ED (figure 2). Overall, 43 patients (28.5%) demonstrated cardiac activity on the initial US in the ED. The mean age of the patients was 65.32 ± 11.68 (35–92) years, and 115 (76.2%) were male. The rate of asystole in initial rhythm was 58.9% (n=89), and the rate of the out-of-hospital cardiac arrest (OHCA) was 84.8% (n = 128).

Table 1 compares the baseline characteristics between cases with ROSC and others. ROSC was achieved in 36 out of 151 (23.8%) patients, twenty patients (13.2%) survived to hospital admission and seven patients (4.6%) survived to hospital discharge. Also, pericardial effusion without tamponade was detected in six patients and right ventricular dilatation in four patients. One of these patients received thrombolytic therapy and survived to hospital discharge. Hypovolemia was detected in five cases. When appropriate management was applied promptly, nine patients were successfully resuscitated (ROSC), and four of them survived to hospital discharge. 

The rate of ROSC in in-hospital cardiac arrest (IHCA) and OHCA was 10 (43.5%) and 26 (20.3%) (p=0.03). Survival to hospital admission was higher for IHCA (30.4% vs 10.2%, p = 0.02) but survival to hospital discharge between IHCA and OHCA was not different (8.7% vs 4.1%, p = 0.29).

The success rate of resuscitation (ROSC) of patients with PEA and asystole rhythm was 43.5% (27 cases) and 10.1% (9 cases), respectively (p<0.001). Among the 36 patients that had cardiac activity at first glance, 24 cases (66.7 %) achieved ROSC and in 115 patients without cardiac activity, 19 cases (16.5%) achieved ROSC (p<0.001). Among the 94 patients in whom no cardiac activity was detected on all scans, only 3 (8.3%) had ROSC and in the other 91 cases (79.1%) the efforts for resuscitation were not successful. None of them survived hospital discharge. Neither age nor gender was a significant predictor of ROSC (Table 1).

***3.2 US findings***

The percentage of patients with cardiac activity on initial US differed between asystole and PEA patients (11.2% vs 53.2%, p<0.001). Twenty-nine cases (46.8%) presenting with PEA had no cardiac activity on initial US (true EMD). On the other hand, 33 cases (53.2%) had cardiac activity (pseudo-EMD). The rates of ROSC were 63.6% for those in pseudo-EMD and 20.7% for those in true EMD. So in patients with PEA rhythm, the presence of cardiac activity during the resuscitation was significantly associated with ROSC (p=0.001).

Six out of 62 patients (9.7%) presenting with PEA had ROSC and survived to hospital discharge. Only one out of 89 cases (1.1%) with asystole as initial rhythm survived to hospital discharge (p=0.02).

The presence of cardiac activity on the first ultrasonography was significantly associated with survival to hospital admission and hospital discharge (Table 2). Among 43 patients with cardiac activity on initial US, six cases (14.0%) survived to hospital discharge, whereas only one out of the 108 (1.0%) patients without cardiac activity on initial US survived to hospital discharge (p = 0.002). The binary logistic regression analysis identified variables that were associated with ROSC (Table 2). Cardiac activity on first scan was associated with ROSC (OR: 6.86, 95%CI: 2.92-16.09), survival to hospital admission (OR: 17.80, 95%CI: 3.95–80.17), and survival to hospital discharge (OR: 17.35, 95%CI: 2.02–148.92) (Table 3).

***3.3 Screening performance characteristics of US in CPR***

The diagnostic performance of US (lack of cardiac activity) for no ROSC, non-survival to hospital admission, and discharge are shown in Table 5. The absence of cardiac activity on US showed a sensitivity and positive predictive value (PPV) of 74.3% (95% CI: 66.4–81.2%) and 99.1% (95% CI: 94.6–99.8%) for non-survival to hospital discharge. None of patients with asystole rhythm and lack of cardiac activity in US survived to hospital discharge (Table 4).

## 4. Discussion:

Point-of-care ultrasound (POCUS) and echocardiography have been suggested for the detection of reversible causes of cardiac arrest during resuscitation in the 2020 update of Advanced Cardiovascular Life Support (ACLS) guidelines, and to assist in the identification of ROSC (13, 14).

In our study, we successfully performed a bedside sonography protocol in keeping with an acceptable 10-second pause for pulse checks during CPR. It is crucial to identify and treat all potential secondary causes of asystole or PEA as rapidly as possible. Potentially reversible causes of hypovolemia, massive PE, and pericardial effusion were detected in 15 cases (9.9%), and four of them survived hospital discharge (2.6%). The rate of reversible causes in our study was low; however, the rate of survival to hospital discharge in these patients was higher than the whole study population (26.7% vs 4.6%,), and this shows the importance of finding reversible causes and promptly treat them to increase patient’s chances of survival. Consistent with our results, Gaspari et al. have demonstrated that PEA with a reversible cause has a higher survival rate to discharge (15.4%) than PEA without a reversible cause (1.3%) (5).

Our study on adult patients with non-traumatic cardiac arrest with non-shockable rhythms indicated that the presence of PEA rhythm and cardiac activity on initial US were associated with ROSC, survival to hospital admission, and hospital discharge. The rate of ROSC was 23.8% in all patients and for those with and without cardiac activity the rates were 66.7% and 16.5%, respectively. Rate of ROSC in previous studies on cardiac arrest patients with cardiac activity range from 24% to 73% (5).

The rate of survival to hospital admission and hospital discharge in patients with cardiac activity was 37.2% and 14.0%, respectively; which is higher than the rate obtained in previous studies (5, 15). The reason for the increase in survival rate can be the treatment of reversible causes found by ultrasound, as well as more efforts to resuscitate patients with cardiac motion and longer CPR of these patients. The overall survival rate to hospital discharge in the present study (4.6%) was comparable to previous studies (5, 15).

Our findings are similar to previous studies that have shown the association of the presence of cardiac activity on initial cardiac US with successful ROSC and survival (5, 6, 15-17). One out of the 108 (1.0%) patients without cardiac activity on initial US survived hospital discharge. Rates of survival to hospital discharge in previous studies in patients without cardiac activity on initial US range from 0% to 10% (18). Chardoli et al. reported that, regardless of the initial rhythm of patients, all of those who did not show cardiac activity in the initial US died (1). One of the studies with the highest survival rates in patients without cardiac activity showed that out of 50 patients without cardiac activity, 5 (10%) survived (3). In another study, of the 530 patients without cardiac activity on initial US, only 3 patients (0.6%) survived hospital discharge (5). A previous meta-analysis and systematic review snapshot reported that the absence of cardiac activity in the US should not be used alone to predict failure of ROSC, with survival to admission rate of 2.4% in patients with cardiac standstill (12). Other studies have reached similar conclusions about the poor prognosis following cardiac arrest associated with the absence of cardiac activity in US (4, 6).

Among 94 patients in whom no cardiac activity was detected on any of the scans, only 3 (8.3%) had ROSC and in the other 91 cases (79.1%) the efforts for resuscitation were not successful. In other words, when the cardiac standstill duration increased to six minutes, no patient lived to hospital discharge. Thus, a cardiac standstill on the serial US may predict non-survival. 

The US is a useful tool for determining pseudo-PEA. Studies show that 42% to 86% of the total PEA patients are pseudo-PEA (19). Out of the 62 patients with PEA, 33 (53.2%) had cardiac activity on initial US (pseudo-EMD). 63.6% of the patients with pseudo-PEA achieved ROSC and there were also higher rates of survival to discharge (15.2%). Chardoli et al. found that 43% of the patients with pseudo-PEA achieved ROSC, whereas no patients with true PEA achieved ROSC (1). Flato et al. showed that 70% of the patients with pseudo-PEA achieved ROSC and 20% of those with true PEA and none of the patients with true PEA survived hospital discharge (20). Cardiac activity in the US had odd ratios of 6.86 for ROSC, 17.80 for survival to hospital admission, and 17.35 for survival to hospital discharge. Lalande et al. reported that cardiac activity in US, compared to its absence, had odd ratios of 16.9 for ROSC, 10.3 for hospital admission, and 8.03 for hospital discharge (21).

The absence of cardiac activity on US showed a sensitivity of 83.5%%, specificity of 66.7%, negative predictive value (NPV) of 55.8%, and PPV of 88.9% for non-ROSC, regardless of the initial rhythm they presented with. In asystole, the PPV of cardiac standstill on US for predicting non-survival to hospital discharge was 100% compared with 96.6% in PEA. In a meta-analysis, ten studies with 1486 participants were included. Presence of cardiac activity on US had a pooled sensitivity of 60.3% (95% CI 38.1–78.9%) and specificity of 91.5% (80.8–96.5%) for ROSC (21). In another study by Bolvardi et al., they found that US had a sensitivity of 73.2%, specificity of 92.2%, NPV of 84.6%, and PPV of 83.7%. However, the inclusion criteria for patients were different in that study (6). In another study by Beckett et al., as a predictor of failure to achieve ROSC, US had a sensitivity of 96.2% and a specificity of 34.0% (22).

**Table 1:** Comparison of baseline characteristics and US findings between patients with return of spontaneous circulation (ROSC) and others

**P value**	**Non-ROSC** ** (n = 115)**	**ROSC ** ** (n = 36)**	**All** ** (n=151)**	**Variables**
				**Age (year)**
0.292	65.89 ± 11.38	63.53 ± 12.59	65.32 ± 11.68	Mean ± SD
				**Gender**
0.654	86 (74.8)	29 (25.2)	115 (76.2)	Male
	29 (80.6)	7 (19.4)	36 (23.8)	Female
				**Initial rhythm**
<0.001	80 (89.9)	9 (10.1)	89 (58.9)	Asystole
	35 (56.5)	27 (43.5)	62 (41.1)	PEA
				**Arrest location**
0.030	102 (79.8)	26 (20.2)	128 (84.8)	OHCA
	13 (56.5)	10 (43.5)	23 (15.2)	IHCA
				**US findings**
<0.001	19 (16.5)	24 (66.7)	43 (28.5)	Cardiac activity on first scan
<0.001	24 (20.9)	33 (91.7)	57 (37.7)	Cardiac activity on any scan
<0.001	1 (0.01)	10 (27.8)	11 (7.3)	Cardiac activity on all scans
<0.001	91 (79.1)	3 (8.3)	94 (62.3)	Cardiac standstill on all scans

Data are presented as mean ± standard deviation (SD) or frequency (%). IHCA: in hospital cardiac arrest, OHCA: out-of-hospital cardiac arrest, PEA: pulseless electrical activity, US: ultrasonography.

**Table 2:** Comparing the studied outcomes between patients with and without cardiac activity on ultrasonography (US) during cardiopulmonary resuscitation (CPR)

**Outcomes**	**Cardiac activity on US**		**P value**
	**With (N=43)**	**Without (N=108)**	
ROSC in ED	22 (51.2)	14 (13.0)	<0.001
Survival to hospital admission	15 (34.9)	5 (4.6)	<0.001
Survival to hospital discharge	6 (14.0)	1 (1.0)	0.002

Data are presented as number (%). ED: emergency department, ROSC: return of spontaneous circulation.

**Table 3:** Predictors of outcomes in studied patients with cardiopulmonary arrest

**Predictors**	**Odds ratio** ^*^	** (95% CI)**	**P value**
**Return of spontaneous circulation (** **ROSC)**			
Initial rhythm (PEA)	7.034	3.097-15.975	<0.001
Initial cardiac activity (yes)	6.857	2.923-16.085	<0.001
Admission status (IHCA)	3.018	1.191-7.649	0.020
**Survival to hospital admission**			
Initial rhythm (PEA)	11.036	3.692-32.986	<0.001
Initial cardiac activity (yes)	17.795	3.950-80.169	<0.001
Admission status (IHCA)	3.870	1.345-11.140	0.017
**Survival to hospital discharge**			
Initial rhythm (PEA)	9.429	1.106-80.407	0.040
Initial cardiac activity (yes)	17.351	2.022-148.922	0.001
Admission status (IHCA)	2.343	0.426-12.874	0.356

IHCA: in hospital cardiac arrest, PEA: pulseless electrical activity, CI: confidence interval. *Odds ratios were calculated using binary logistic regression analysis.

**Table 4:** Screening performance characteristics of ultrasonography during cardiopulmonary resuscitation for predicting the return of spontaneous circulation (ROSC), survival to hospital admission, and survival to hospital discharge

**Characters**	**ROSC**	**Hospital admission**	**Hospital discharge**
**Overall**			
Sensitivity	83.5 (75.4-89.8)	59.1 (43.2-73.7)	74.3 (66.4-81.2)
Specificity	66.7 (49.0-81.4)	83.3 (58.6-96.4)	85.7 (42.1-99.6)
PPV	88.9 (83.3-92.7)	89.7 (75.0-96.1)	99.1 (94.6-99.8)
NPV	55.8 (44.1-66.9)	45.5 (35.6-55.7)	95.4 (90.7-98.1)
Accuracy	79.5 (72.1-85.6)	66.1 (53.0-77.7)	74.8 (67.1-81.5)
**Pulseless electrical activity**			
Sensitivity	65.7 (47.8-80.9)	59.1 (43.2-73.7)	50.0 (36.3-63.7)
Specificity	77.8 (57.7-91.4)	83.3 (58.6-96.4)	83.3 (35.9-99.6)
PPV	79.3 (65.5-89.0)	89.7 (75.0-96.1)	96.6 (82.1-99.4)
NPV	63.6 (51.5-74.3)	45.5 (35.6-55.7)	15.2 (10.3-21.8)
Accuracy	71.0 (58.1-81.8)	66.1 (53.0-77.7)	53.2 (40.1-66.0)
**Asystole **			
Sensitivity	91.2 (82.8-96.4)	89.7 (81.3-95.2)	89.8(81.5-95.2)
Specificity	33.3 (7.5-70.1)	50.0 (1.3-98.7)	100
PPV	92.4 (88.4-95.1)	98.7 (95.1-99.7)	100
NPV	30.0 (11.8-57.8)	10.0 (2.4-33.6)	0.1(0.1-0.2)
Accuracy	85.4 (76.3-92.0)	88.8 (80.3-94.5)	89.9(81.7-95.3)

Data are presented with 95% confidence interval. PPV: Positive predictive value, NPV: negative predictive value.

##  5.Limitations

The study sample size was relatively small. The most prominent limitation of this study, however, was potentially increased risk of bias with regard to lack of blinding for US results, and that patients with cardiac activity showed longer resuscitation times. The next limitation, presumably, is that the protocol used in this study has not been compared with other protocols. 

## 6. Conclusion:

In non-traumatic cardiac arrest patients with non-shockable rhythms, bedside US is of great importance in predicting ROSC. The presence of PEA rhythm and cardiac activity on initial US were associated with ROSC, survival to hospital admission, and hospital discharge. When the cardiac standstill duration increased to six minutes, no patient survived hospital discharge. So, the absence of cardiac activity on serial US could imply that prolonged resuscitation may not provide measurable benefit.

## 7. Declarations:

### 7.1 Acknowledgment

The authors appreciate the insightful cooperation of the staff of the emergency departments of Al-Zahra hospital and Kashani hospital, Isfahan, Iran.

### 7.2 Authors' contribution

Surgical and Medical Practices and Concept:  B.M., F.H., M.Z., R.A., M.N.I., Design:  B.M., F.H., R.A., Data Collection or Processing: B.M., F.H., M.Z., R.A., M.N.I., Analysis or Interpretation: F.H., B.M., Literature Search and Writing: B.M., F.H., M.Z., R.A., M.N.I., All authors approved the final version.

### 7.3 Conflicts of interest

The authors declare no conflict of interests.

### 7.4 Funding and supports

This research was performed with the support of Isfahan University of Medical Sciences.
